# Supplementary material for: Association between oral microbiome and seven types of cancers in East Asian population: a two-sample Mendelian randomization analysis
Source: Front Mol Biosci. 2023 Nov 21;10:1327893. doi: 10.3389/fmolb.2023.1327893 (PMC10702768; doi:10.3389/fmolb.2023.1327893)
Supplement: Supplementary file 1 [file DataSheet1.ZIP › Supplementary Materials/MR plots of saliva/saliva═╝/Pancreatic cancer/pheno.1353_to_pancreatic cancer_leave_one_out.pdf]

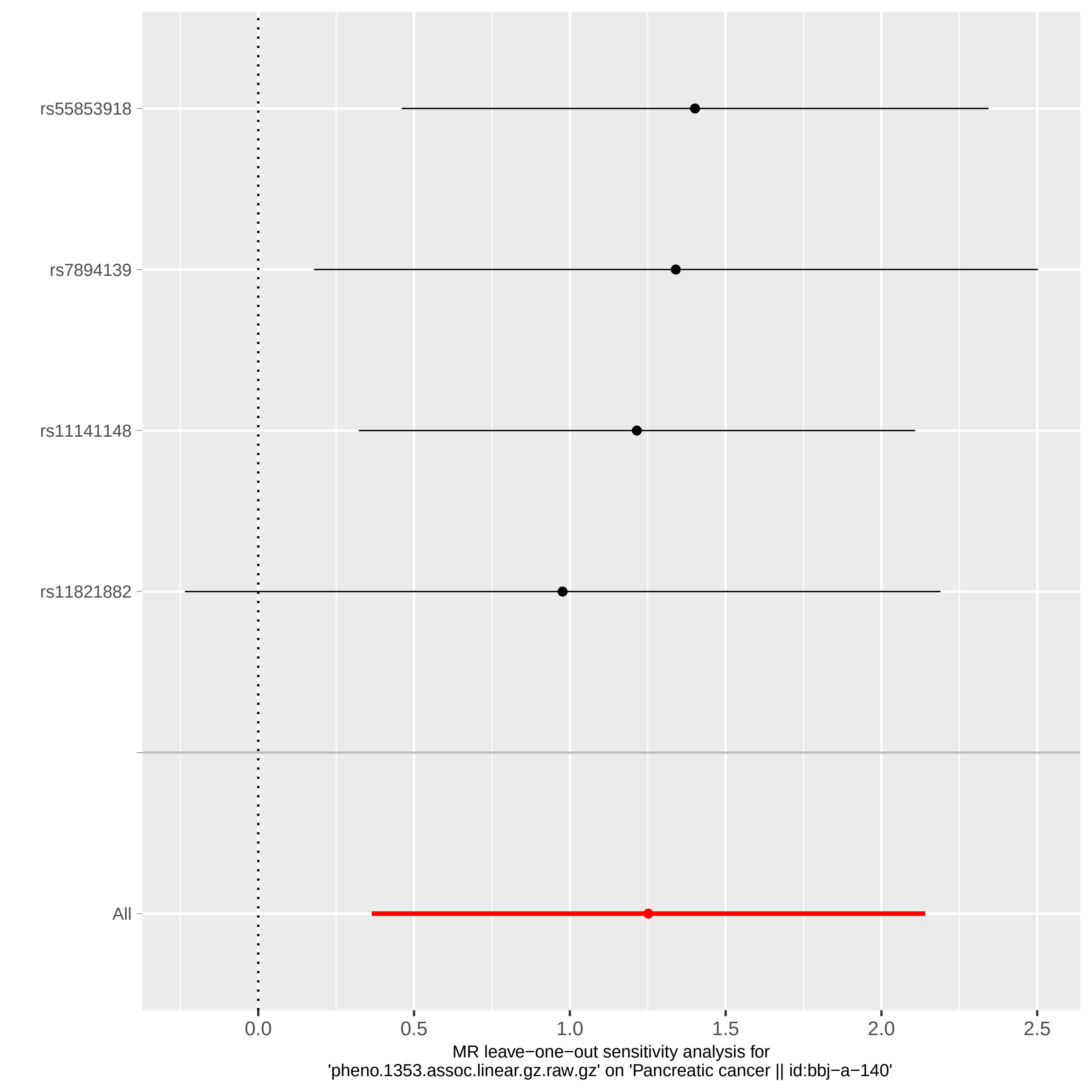

rs55853918
rs7894139
rs11141148
rs11821882
All
0.0
0.5
1.0
1.5
2.0
2.5
MR leave−one−out sensitivity analysis for
'pheno.1353.assoc.linear.gz.raw.gz' on 'Pancreatic cancer || id:bbj−a−140'
